# Effects of Dietary Inclusion Level of Microwave-Dried and Press-Defatted Black Soldier Fly (*Hermetia illucens*) Larvae Meal on Productive Performance, Cecal Volatile Fatty Acid Profile, and Egg Quality in Laying Hens

**DOI:** 10.3390/ani11061486

**Published:** 2021-05-21

**Authors:** Seol-Hwa Park, Hye-Ran Kim, Youl-Chang Baek, Chae-Hwa Ryu, Sang-Yun Ji, Jin-Young Jeong, Minji Kim, Hyunjung Jung, Byeonghyeon Kim

**Affiliations:** Animal Nutrition & Physiology Team, National Institute of Animal Science, Rural Development Administration, Wanju 55365, Korea; shwa6560@korea.kr (S.-H.P.); ococ1004@korea.kr (H.-R.K.); chang4747@korea.kr (Y.-C.B.); chryu0629@korea.kr (C.-H.R.); syjee@korea.kr (S.-Y.J.); jeong73@korea.kr (J.-Y.J.); mjkim00@korea.kr (M.K.); hyjjung@korea.kr (H.J.)

**Keywords:** black soldier fly, egg quality, fatty acid profile, heavy metal, insect meal, laying hen

## Abstract

**Simple Summary:**

The microwave drying method is convenient in terms of time efficiency, space, and low operational cost compared with the conventional air-drying method. For the same reasons, the microwave drying method has the potential for manufacturing insect meals that could be an alternative protein source in poultry. Therefore, we evaluated the productive performance, cecal volatile fatty acid (FA) profile, and egg quality in laying hens fed on microwave-dried *Hermetia illucens* larvae meal (HILM) at two different substitution levels (2% and 4%) of soybean meal. Similar productive performance with no negative effects on the nutritional and physical quality of eggs was observed from the study, indicating that microwave-dried HILM can be a potential ingredient in the diets of laying hens. However, further research is needed in improving the manufacturing process for better bioavailability of HILM and improved FA quality of eggs.

**Abstract:**

Black soldier fly (*Hermetia illucens*) larvae meal (HILM) is a promising alternative to soybean meal (SBM). However, little information is available on the effect of microwave-dried HILM as a dietary protein source in the diets of laying hens. We studied the effect of dietary inclusion level of microwave-dried HILM on productive performance, cecal volatile fatty acid profile, egg quality, overall fatty acid profile, and heavy metal residues of the egg in laying hens. A total of 144 laying hens (25-week-old) were randomly assigned to three dietary groups (eight replicates and six birds/cage): a control diet, and two experimental diets in which SBM was replaced with 2% HILM (2HILM) and 4% HILM (4HILM). The laying hens that fed the HILM showed satisfactory results in productive performance and egg quality. Branched-chain fatty acid levels increased linearly (*p* < 0.001) with dietary treatment in the cecal digesta. Total monounsaturated fatty acid increased linearly (*p* < 0.01), while total polyunsaturated fatty acid decreased linearly (*p* < 0.01) in the eggs by dietary treatments. Heavy metals, magnesium, zinc, and aluminum were increased linearly with dietary treatment; however, undesirable heavy metals were under permissible levels. Thus, microwave-dried HILM could be a possible alternative to SBM in the diets of laying hens; however, improvements in fatty acid profile are needed.

## 1. Introduction

Soybean meal (SBM) is the main dietary protein source in poultry diets, however, the price of SBM has been soaring. For this reason, alternative dietary protein sources with comparable protein and amino acid content are needed to cope with the limited supply of soybean and increased demand for human consumption [1,2]. Moreover, managing organic waste by the human population is also a major issue [2]. Organic waste can be decomposed by *Hermetia illucens* larvae (HIL) and recycled to dietary protein and fat sources [3,4,5]. However, the potential risk of heavy metal accumulation in the larval body from contaminated rearing substrates should be monitored [6].

HIL has the potential to be an ingredient in poultry diets and HIL meal (HILM) has been investigated as an alternative protein source in poultry diets [1,7] as it contains similar or higher valuable protein and amino acid content than SBM [7,8]. However, with increased inclusion of HILM in broilers, chitin in the exoskeleton of larvae negatively impacted crude protein digestibility, and the growth performance decreased linearly, showing that low inclusion level of HILM was appropriate for diets [9,10]. Furthermore, the high level of inclusion of HILM showed negative effects on laying performances such as lay percentage, feed intake, egg mass, and feed conversion ratio (FCR) [11,12]. In contrast, chitin has been reported to have a positive effect by increasing cecal volatile fatty acid (VFA) concentration and decreasing blood cholesterol level [13,14]. Thus, proper ingestion levels of chitin in the HILM should be considered.

In previous studies, the focus was on the effect of HILM on the fatty acid (FA) profile of animal products such as meats and eggs [7,13,14]. In comparison to the FA profile of SBM, HILM contains high levels of saturated fatty acid (SFA) and low levels of polyunsaturated fatty acid (PUFA) [13]. It is reported that the FA profile differences between HILM and SBM are reflected in chicken meats and eggs [7,15,16]. Furthermore, a positive effect of pigment in HILM on yolk color was observed, making it appear redder [7,15].

Apart from good nutrient composition and chitin effects, the drying process is also important for the chemical composition of nutrients [17]. Particularly, the microwave drying method, which is commonly used for food processing, manages moisture content in the insect larvae and produces time and energy-efficient products [17,18]. Nevertheless, the effects of microwave-dried HILM on the diets of laying hens are not yet clear. In this study, SBM was replaced with microwave-dried HILM as a dietary protein source in the diets of laying hens to evaluate productive performance, cecal VFA profile, egg quality, FA profile, and heavy metal residue in the eggs.

## 2. Materials and Methods

### 2.1. Animals, Diets, and Insect Meal

The experiment was conducted in the poultry facility of the National Institute of Animal Science of South Korea, and approved by the Institutional Animal Care and Use Committee of the Rural Development Administration (No. NIAS-2020-498). A total of 144 Hy-line Brown hens (25-week-old, average live weight 1.95 ± 0.05 kg standard deviation) were equally allotted to three dietary treatments (48 hens per treatment) and then distributed into eight cages (six hens/cage) for each group. The control group (CON) was fed a diet based on corn and SBM, and the other two groups were supplemented with 2% and 4% of microwave-dried and press-defatted HILM (2HILM and 4HILM, respectively). All groups were fed isoproteic and isoenergetic diets with differing ratios of other ingredients such as corn and soybean oil. The diets were formulated to meet or exceed the requirements of the laying hens [19] and were provided ad libitum throughout the trial. The ingredients and chemical composition of the experimental diets are presented in Table 1.

**Figure 1:**
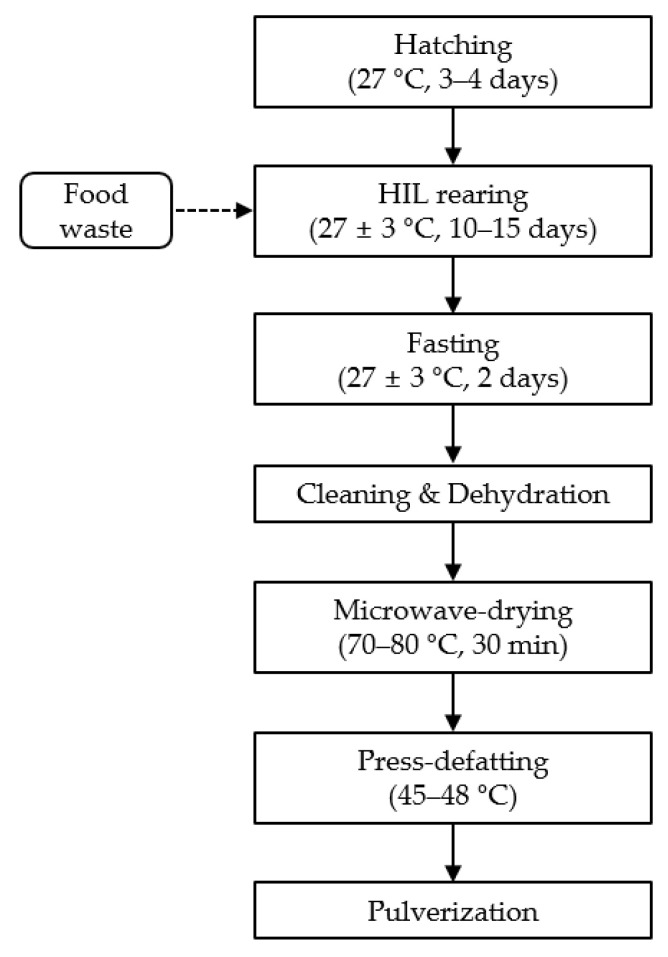
Schematic presentation of manufacturing of *Hermetia illucens* larvae meal (HILM).

The schematic overview of the manufacturing process of HILM is presented in Figure 1. HIL eggs hatched after three to four days at approximately 27 °C; HIL were then reared on household food waste for 10–15 days at 27 ± 3 °C. The HIL were made to undergo fasting for two days to remove waste from their bodies to be rendered suitable as a feed ingredient. Before manufacturing of HILM, the HIL were cleaned, dehydrated, and dried using a microwave-drying oven at 70–80 °C for 30 min. The dried HIL were press-defatted at 45–48 °C using a cold press oil machine (NF-80; Karaerler, Ankara, Turkey) to prevent nutritional loss and denaturation. The defatted HIL were then pulverized to mix evenly with other ingredients.

### 2.2. Productive Performance

The live weight of hens was recorded at the beginning and the end of the experiment. Egg collection to record productive performance started after one week of adaptation to the new diets. The number of eggs produced and the weight of each egg were recorded daily from 26 to 33 weeks of age per replicate to calculate the lay percentage and average egg weight. Feed intake was also measured per replicate to calculate daily feed intake per hen. For each replicate, the egg mass was calculated by multiplying egg weight with lay percentage and the FCR was calculated in grams as a quantity of feed intake divided by the weight of egg produced.

### 2.3. Slaughtering and Volatile Fatty Acid Analysis

At 33 weeks of age, one hen was randomly selected per cage (eight laying hens per treatment) and slaughtered. The cecal digesta samples were collected and stored at −80 °C until analysis. Approximately 1 g of cecal digesta sample was thawed and diluted with 1 mL of distilled water and centrifuged at 5000× *g* at 4 °C for 10 min. The supernatant was transferred and 200 µL of 25% metaphosphoric acid was added, and then centrifuged at 5000× *g* for 10 min. The clear supernatant was analyzed to measure the concentrations of acetate, propionate, butyrate, isobutyrate, valerate, and isovalerate by gas chromatography (6890N; Agilent Technologies, Waldbronn, Germany) with a Nukol^TM^ fused silica capillary column (15 m × 0.53 mm × 0.5 µm film thickness; Supelco Inc., Bellefonte, PA, USA). The oven temperature was held at 110 °C for 1 min, increased to 125 °C (heating rate: 10 °C/min) in 1.5 min, then increased again to 200 °C (heating rate: 10 °C/min) in 7.5 min, and held at this temperature for three min. Each sample was injected (injection volume: 5 μL) at 250 °C with a 55:1 split ratio using hydrogen as a carrier gas with a flow of 11 mL/min. The temperature of the flame ionization detector was 250 °C with a hydrogen flow of 30 mL/min, an air flow of 300 mL/min, and nitrogen was used as the makeup gas at 25 mL/min.

### 2.4. Chemical Analyses of Insect Meal, Soybean Meal, and Eggs

The chemical composition of two protein sources (HILM and SBM) was determined based on previously described methods [20]. Furthermore, the same insect meal and SBM were used in this study, as shown in our previous study [21].

Three eggs were homogenized into one adequate replicate to perform analysis of the proximate composition, cholesterol content, and FA profiles of whole eggs. The analysis was performed with the homogenized egg samples (eight replicates of three eggs per cage) at the end of the trial. The proximate composition of the eggs was analyzed according to the AOAC [20] methods. The cholesterol content of the egg was analyzed using gas chromatography according to Ahn et al. [22].

The FA profiles of the HILM, SBM, and eggs were determined following the method described by Kim et al. [21]. The lipid extraction of the samples was performed as described by Folch et al. [23]. The samples were trans-methylated using a methanolic solution of H_2_SO_4_ (4%), and the FA methyl esters (FAME) were determined using gas chromatography (Star 3600; Varian Technologies, Palo Alto, CA, USA), equipped with an Omegawax 205 fused-silica bond capillary column (30 m × 0.32 mm × 0.25 µm film thickness). The oven temperature was held at 50 °C for 1 min and increased until 200 °C (heating rate: 25 °C/min). Each sample was injected into the injection ports with the temperatures of the injector and the detector at 250 °C and 260 °C, respectively. The carrier gas was nitrogen at a constant flow of 1 mL/min. The FA composition of the samples was expressed as the percentage of total detected FAME.

### 2.5. Egg Quality Characteristics

Egg quality analysis was performed with 16 eggs per treatment (eight replicates of two eggs per cage), which were randomly collected at the end of the study. Eggshell strength (kgf/cm^2^) was measured by a texture analyzer (TAHDi 500; Stable Micro System, Godalming, UK) and eggshell thickness (mm) was measured at three different locations (top, middle, and bottom) using a dial pipe gauge (model 7360, Mitutoyo Corporation, Kawasaki, Japan). Eggshell color was analyzed using an eggshell color fan (Samyangsa, Kangwon-do, Korea) and yolk color was evaluated by comparison with the Roche color fan (Hoffman-La Roche, Basel, Switzerland). Haugh unit (HU) values were calculated from the measurements of albumen height (H) and egg weight (W) using the following equation:

HU = 100 log (H − 1.7W^0.37^ + 7.6)

as described by Eisen et al. [24].

### 2.6. Heavy Metal Analysis

After analysis of the proximate composition, cholesterol content, and FA profiles of the eggs, the analysis for heavy metal concentrations of the egg samples and the HILM was performed. The samples were digested using nitric acid and hydrogen peroxide in a microwave digestion system. The digested samples were filtered and then transferred to acid-cleaned tubes. The concentrations of heavy metal in the samples were determined using inductively coupled plasma-mass spectrometry (Agilent 7700×; Agilent Technologies, Santa Clara, CA, USA).

### 2.7. Statistical Analysis

Caged and individual laying hens were the experimental units for statistical analysis of performance and VFA concentration, while the homogenized egg sample was an experimental unit for physicochemical analysis of the eggs. The data were processed using the SAS version 9.4 GLM procedure and Tukey’s multiple range test to determine the differences among treatments [25]. In addition, the data were further analyzed using the orthogonal contrast analysis for linear and quadratic effects between the means [25]. The results are presented as mean ± standard error of the means. Significance was declared at *p* < 0.05 and tendency was considered for *p* < 0.10.

## 3. Results

### 3.1. Chemical Composition of Larvae and Soybean Meal

As described in our previous study [21], the HILM had higher crude protein, acid detergent fiber (ADF), and ADF-linked protein contents than SBM (61.24% vs. 45.76%, 11.24% vs. 9.50%, 5.54% vs. 3.46%, respectively). The chitin level was calculated according to a method by Marono et al. [9] and it was approximately 5.70%. The FA profiles of the HILM showed that total SFA was higher than SBM (55.15% vs. 22.89%). In contrast, total PUFA was lower in the HILM than SBM, as shown in our previous study [21].

### 3.2. Changes in Body Weight and Productive Performance

The body weight (BW) changes and productive performance results of laying hens fed with high levels of HILM are presented in Table 2 and Table 3. The BW changes were not affected by the dietary treatments in Table 2. Table 3 shows that the lay percentage decreased linearly (*p* = 0.022) with the inclusion level of HILM; however, there was no significant difference between the CON and 2HILM groups. Dietary treatments also did not affect the egg weight, feed intake, and egg mass. The FCR did not differ among the dietary treatments during the entire experimental period.

### 3.3. Concentrations of Volatile Fatty Acids in Cecal Digesta

The cecal VFA concentration results of laying hens are summarized in Table 4. The absolute values of acetate, isobutyrate, and valerate linearly increased (*p* < 0.01) with the inclusion level of HILM and were significantly higher (*p* < 0.05) in 4HILM than that of the CON group. There was a tendency to increase (*p* = 0.057) in isovalerate concentration with the inclusion level of HILM. The concentration of branched-chain fatty acid (BCFA; isobutyrate, valerate, and isovalerate) linearly increased (*p* = 0.0003) and that of total short-chain fatty acid (SCFA) tended to increase (*p* = 0.057) with the dietary treatments. Furthermore, the concentration of BCFA in the 4HILM group was significantly higher (*p* = 0.001) than that of the CON group. The relative values of acetate and valerate linearly increased (*p* < 0.01), while that of propionate linearly decreased (*p* < 0.0001) with the dietary treatment. The 4HILM group showed a higher (*p* < 0.05) relative value of acetate and valerate, and lower propionate (*p* < 0.0001) value in 4HILM than that of the CON group.

### 3.4. Physical and Chemical Quality of Eggs

The effects of HILM on the physical traits of eggs are presented in Table 5. The yolk color was significantly higher (*p* = 0.037) in the 4HILM group than that of the CON group and linearly increased (*p* = 0.019) with the inclusion level of HILM. However, there were no differences in the HU, strength, thickness, and color of the eggshell.

Table 6 shows the effect of the dietary treatments on the proximate composition and cholesterol content of eggs. No significant differences were observed in proximate composition such as water, protein, lipids, and ash. However, the cholesterol content tended to decrease (*p* = 0.061) by dietary treatments.

### 3.5. Fatty Acid Profiles of Eggs

The effects of the dietary HILM on the FA profiles of eggs are presented in Table 7. The myristic and palmitic acids linearly increased (*p* < 0.05), whereas stearic acid linearly decreased (*p* = 0.011). Furthermore, a quadratic response was observed (*p* = 0.042) for palmitic acid with the highest observed for the 2HILM group. However, total SFA content was not affected by dietary treatments. The oleic acid linearly increased (*p* = 0.006) and was higher (*p* = 0.020) in the 4HILM group than in the CON group. The total monounsaturated fatty acid (MUFA) linearly increased (*p* = 0.003) and was higher (*p* = 0.010) in the 4HILM group than in the CON group. The linoleic acid, docosahexaenoic acid (DHA), and total PUFA linearly decreased (*p* < 0.05), with the linoleic acid and total PUFA values lower (*p* < 0.05) in the 4HILM group than in the CON group. The *n*-6 and *n*-3 FAs linearly decreased (*p* < 0.05) and were lower (*p* < 0.05) in the 4HILM group than in the CON group.

### 3.6. Heavy Metal Concentration in Insect Meal and Eggs

Table 8 and Table 9 show the concentrations of essential elements and undesirable substances in HILM and eggs. The concentrations of undesirable substances, fluorine (F), arsenic (As), lead (Pb), mercury (Hg), and cadmium (Cd) were under the permissible limit for animal feed [26]. The concentrations of magnesium (Mg), zinc (Zn), and aluminum (Al) linearly increased (*p* < 0.01) with the dietary inclusion level of HILM and were higher (*p* < 0.01) in the 4HILM group than in the CON group. However, the Mg and Zn were not applicable and Al was below the permissible limit for eggs [27]. As, Pb, Hg, and Cd were also below the permissible limits for eggs [28,29].

## 4. Discussion

Appropriate energy and protein requirements are necessary for optimal egg production without increasing BW and plumpness during the laying period. In particular, an optimal level of crude protein is important to maintain growth and maximize productive performance in poultry [30]. Hence, to be considered as a suitable protein ingredient in poultry, protein digestibility is an important factor [14]. Although insects are a natural diet of poultry and HIL, being a high protein and amino acid source, is comparable to SBM [3,31], the exoskeletal chitin of larvae can negatively affect the protein digestibility [11,32].

Despite the nutritional concerns of HILM, our results showed that the dietary inclusion of HILM did not impair the BW changes during the experimental period. In laying quail diets, the addition of HILM up to 15% did not affect growth performance [7,31]. Laying pullets that fed on a 7.5% inclusion level of HILM during the experimental period (19 to 27 weeks of age) showed higher BW at 27 weeks [12] and those that fed on a 15% inclusion level of HILM during the experimental period (28 to 43 weeks of age) had higher BW at 43 weeks [33]. However, total replacement of SBM with HILM in the diets of Lohmann Brown Classic laying hens (24 to 45 weeks of age) decreased weight gain [11]. In contrast, in laying hens (Hy-line Brown), the inclusion levels (7.3% and 14.6%) of HILM did not show the difference in BW at 40 weeks [14]. In this study, laying hens that fed on HILM showed no negative effects of weight gain during the experimental period and the different BW results might be due to different inclusion levels of insect meal, experimental periods, species, or ages.

The productive performance of laying hens fed with HILM showed that there were no negative effects on FCR results; however, the lay percentage decreased in the 4HILM group. It was reported in a previous study that total replacement of SBM with HILM in a laying hen diet decreased laying performance by decreasing lay percentage, egg weight, and egg mass [11]. In contrast, it was reported that soybean cake could be replaced with HILM in layer diets without negative effects on productive performance and health [34]. The contrasting results can be attributed to different starting ages (24 and 64 weeks of age, respectively), experimental periods (21 and 10 weeks, respectively), or the dark coloration of insect meal diets [11]. It is suggested that the lower feed intake was ascribed to the darker color of the HILM diet than that of the SBM diet [11]. In another study, it is suggested that SBM can be substituted with microwave-dried HILM (<7%) in broiler diets [21]. The suggested substitution level (7%) of the microwave-dried HILM was lower than the results (<10% in broiler diets), which were reported in previous studies [10,16]. In laying hens (19 to 27 weeks of age), it has been reported that a 7.5% inclusion level of HILM showed negative effects on FCR [12]. Moreover, the FCR of older laying hens (28 to 43 weeks of age) that fed on HILM (0–15%) was also high [33]. In this study, FCR was not affected by dietary treatments and this might be due to low inclusion levels of HILM and the short experimental period. Therefore, the low inclusion level of microwave-dried HILM seems to be appropriate for productive performance in younger laying hens.

The positive effects of chitin on gut health were confirmed by a linear increase in cecal BCFA level and a tendency to a higher total SCFA in the 4HILM group. In this study, total SCFA tended to increase with the inclusion level of HILM and increased by about 54% in the 4HILM group. Similar to our result, Cutrignelli et al. [35] showed increased total VFA concentration (>36.8%) in HILM-fed broilers. In a previous study, the cecal content of butyrate in laying hens also increased with HILM [35], in contrast, the concentration of butyrate was not affected in our result. Moreover, the concentration of the total BCFA in the 4HILM group drastically increased (approx. 89%) than in the CON group. Although the reason is unclear, it could be ascribed to different chemical compositions of the microwave-dried HILM compared to previous studies that used a different manufacturing process. The alteration of the chemical structure of the protein particle in the microwave-dried HILM might have contributed to lower protein digestibility [36]. The microwave drying method makes the protein particle more compact by polymerization reaction [36]. Furthermore, the characteristics of chitin combined with other nutrients in insect meals render it difficult to digest [9,14]. In this study, laying hens on 2HILM and 4HILM feeds ingested approximately 0.14 and 0.29 g/d of chitin, respectively, according to the method by Marono et al. [9]. Increasing the ingestion level of chitin could affect digestibility, and therefore appropriate inclusion level of HILM should be considered for protein digestibility [14]. Hence, proteolytic fermentation of the undigested protein in the cecum may have affected the concentrations of the BCFAs [10]. The increase in the intestinal length of layers fed insect meal by a compensatory mechanism for increasing nutrient absorption surface and digestibility efficiency corroborates the availability of proteolytic fermentation in the cecum [14,37].

In this study, the egg quality parameters except for the yolk color were not different among the treatment groups. In a previous study, the eggshell thickness was increased by the inclusion levels (5% and 7.5%) of HILM [12]. A similar result was reported in laying quails’ eggs where the physical parameters such as shell thickness improved [7]. It was also suggested that the hindgut fermentation of chitin may have contributed to an increase in mineral absorption such as calcium [12]. However, in our study, differences in physical traits of eggs were not observed and the results were due to low chitin ingestion levels in the 2HILM and 4HILM groups. The noticeable alteration was increased intensity of yolk color in the 4HILM group and a common result was reported in previous studies [7,12,15]. The alteration of yolk color is related to carotenoids such as β-carotene and lutein in the HILM affecting the yolk color [7,15]. In a previous study, total carotenoid content was 2.15 mg/kg in the HIL [15]. Furthermore, 5% inclusion level of HILM in laying pullet diets improved yolk color [12]. These results indicate that eggs from laying hens that were fed HILM can contribute to consumer acceptability by enhancing the yolk color.

The proximate composition of eggs from laying hens fed on HILM did not differ among dietary treatments and suggests that it can be considered as a feed ingredient. Secci et al. [15] also reported that total replacement (inclusion level of 17%) of SBM with HILM had no negative effect on the proximate composition of eggs in laying hens. In contrast, the protein content of eggs from laying quails fed 15% HILM in a diet was decreased [7]. They suggested that the negative effect of chitin on nutrient digestibility resulting in lower protein availability could have affected the protein content in the eggs [7]. Furthermore, in line with the finding of Secci et al. [15], we also observed that cholesterol levels tended to decrease in eggs from laying hens fed on HILM. This reduction in cholesterol level can be ascribed to lower serum cholesterol in laying hens that fed on HILM, in addition to chitin contributing to decreased lipid absorption by binding to the lipids and fatty acids [11,14]. Furthermore, the bile acid-binding capacity of the chitin could inhibit bile reabsorption and enhance cholesterol excretion by hypocholesterolemia, resulting in lower cholesterol levels in the blood [38,39]. However, cholesterol levels in laying quails’ eggs and growing quail breast meats were not affected by the HILM [7,40]. This discrepancy might be due to different species.

The FA profiles of the larvae depend on their rearing system and the alteration of FA profiles in laying hen eggs reflected the FA profiles of the HILM. In this study, we observed increased total MUFA and decreased total PUFA. A similar result was also reported, where 10% and 15% inclusion levels of the HILM in laying quails’ diets increased total MUFA and decreased total PUFA in eggs, and increased total SFA content on the HILM fed groups [7]. However, in our findings, total SFA content was not affected by dietary treatments and the discrepancy meant that inclusion levels in the 2HILM and 4HILM groups were too low to change the SFA content in the eggs. Moreover, the increase of the total MUFA content was associated with the SFA content and was contributed by elongation and desaturation of SFA to the MUFA [7,41]. There was also a reduction in important precursors (linoleic and linolenic acids) with inclusion levels of the HILM, which led to decreased *n*-6 and *n*-3 FAs such as DHA and eicosapentaenoic acid in the eggs [7,42].

Heavy metal concentrations in the HILM and animal products have to be monitored due to the residues of undesirable substances. The detrimental heavy metals can be accumulated in the HIL body from their contaminated feeding media [5,43,44]. In our trial, undesirable substances such as F, As, Pb, Hg, and Cd in the HILM were under permissible limits [26]. Furthermore, the hazardous heavy metals in the eggs did not exceed the permissible limits [27,28,29]. Although the concentrations of Mg and Zn increased in the 4HILM group compared to the CON group, they were not applicable for restriction. These results indicate that in terms of safety, the HILM is suitable as a feed ingredient for animal diets.

## 5. Conclusions

In conclusion, microwave-dried HILM can be considered as a possible alternative ingredient to SBM in laying hen diets. Although lay percentage was lower in the 4HILM group than in the CON group, our data showed similar productive performance such as feed intake, egg mass, and FCR. Furthermore, the egg quality results among the dietary treatments were not different. We also observed improved cecal SCFA concentration, egg cholesterol level, and yolk color. However, an improvement in the FA profiles of eggs is necessary by modulating the rearing conditions of larvae for consumer acceptance. Further investigations are required for the availability of a higher substitution level by improving the manufacturing process considering the negative effects of the microwave-dried HILM on lay percentage.

## Figures and Tables

**Table 1:** Ingredients and chemical composition of the experimental laying hens’ diets containing different levels of *Hermetia illucens* larvae meal (HILM) ^1^.

Item	Dietary Treatment		
	CON	2HILM	4HILM
Ingredients, %			
Corn	59.42	61.03	62.56
Soybean meal, 45%	24.31	21.28	18.34
HILM	0.00	2.00	4.00
Wheat bran	2.00	2.00	2.00
Soybean oil	4.00	3.65	3.30
Dicalcium phosphate	1.04	1.03	1.01
Limestone	8.56	8.35	8.14
L-lysine, 78%	0.02	0.02	0.02
DL-methionine	0.15	0.14	0.13
Salt	0.20	0.20	0.20
Vitamin-mineral premix ^2^	0.30	0.30	0.30
Calculated composition			
ME, kcal/kg	2814	2814	2815
Lysine, %	0.84	0.84	0.84
Methionine, %	0.41	0.41	0.41
Calcium, %	3.80	3.80	3.80
Total phosphorus, %	0.60	0.60	0.60
Analyzed composition, %			
Crude protein	15.81	15.84	15.90
Crude fat	6.41	6.64	6.73
NDF	7.95	8.85	8.70
ADF	3.14	3.17	3.44
Ash	12.92	11.89	13.10

CON, control diet; 2HILM and 4HILM are diets corresponding to 2% and 4% HILM inclusion levels, respectively; ME, metabolizable energy; NDF, neutral detergent fiber; ADF, acid detergent fiber. ^1^ As-fed basis. ^2^ Supplied per kilogram of diet: vitamin A 1,600,000 IU; vitamin D_3_ 300,000 IU; vitamin E 800 IU; vitamin K_3_ 132 mg; vitamin B1 97 mg; vitamin B2 500 mg; vitamin B6 200 mg; vitamin B12 1.2 mg; nicotinic acid 2000 mg; pantothenic acid 800 mg; folic acid 60 mg; choline chloride 35,000 mg; Mn 12,000 mg; Zn 9000 mg; Fe 4000 mg; Cu 500 mg; I 250 mg; Co 100 mg; Se 50 mg.

**Table 2:** Changes in body weight (BW) of laying hens during the trial (*n* = 8 cages/treatment).

Item	Dietary Treatment			SEM	*p*-Value		
	CON	2HILM	4HILM		Diet	Contrast Analysis	
						Linear	Quadratic
Initial BW, g	1951.11	1949.06	1944.34	23.66	0.980	0.849	0.964
Final BW, g	2031.31	2104.68	2068.18	40.83	0.497	0.552	0.298
Weight gain, g	80.20	155.62	123.87	22.46	0.112	0.211	0.076

CON, control diet; 2HILM and 4HILM are diets corresponding to 2% and 4% HILM inclusion levels, respectively; SEM, standard error of the means.

**Table 3:** Effect of the dietary *Hermetia illucens* larvae meal (HILM) inclusion level on the productive performance of laying hens.

Item	Dietary Treatment			SEM	*p*-Value		
	CON	2HILM	4HILM		Diet	Contrast Analysis	
						Linear	Quadratic
N. of laying hens	48	48	48				
N. of replicated cages	8	8	8				
Lay, %	97.84 ^a^	97.73 ^a^	96.06 ^b^	0.51	0.038	0.022	0.226
Egg weight, g	61.24	60.44	61.48	0.59	0.445	0.777	0.219
FI, g/d/hen	122.38	123.07	125.45	1.98	0.527	0.286	0.731
Egg mass	59.90	59.04	59.04	0.61	0.520	0.326	0.567
FCR	2.04	2.09	2.13	0.03	0.217	0.084	0.989

CON, control diet; 2HILM and 4HILM are diets corresponding to 2% and 4% HILM inclusion levels, respectively; SEM, standard error of the means; FI, feed intake; FCR, feed conversion ratio. ^a,b^ Values with different superscripts in the same row are significantly different (*p* < 0.05).

**Table 4:** Effect of the dietary *Hermetia illucens* larvae meal (HILM) inclusion level on the volatile fatty acid (VFA) concentrations in the cecal contents of laying hens.

Parameter	Dietary Treatment			SEM	*p*-Value		
	CON	2HILM	4HILM		Diet	Contrast Analysis	
						Linear	Quadratic
Absolute value, mmol/g							
Acetate	45.09 ^b^	59.95 ^ab^	78.48 ^a^	6.33	0.005	0.001	0.815
Propionate	14.27	16.53	15.85	1.83	0.676	0.548	0.521
Butyrate	12.73	14.03	14.41	3.45	0.937	0.733	0.915
Isobutyrate	0.98 ^b^	1.49 ^ab^	1.80 ^a^	0.20	0.029	0.009	0.687
Valerate	2.56 ^b^	3.33 ^b^	5.58 ^a^	0.53	0.002	0.001	0.268
Isovalerate	1.92	2.75	2.94	0.30	0.057	0.025	0.397
BCFA ^1^	5.46 ^b^	7.57 ^ab^	10.33 ^a^	0.79	0.001	0.0003	0.742
Total SCFA ^2^	77.55	98.07	119.07	11.43	0.057	0.018	0.986
Relative value, % of total VFAs							
Acetate	60.02 ^b^	60.79 ^b^	66.15 ^a^	1.43	0.013	0.007	0.205
Propionate	18.74 ^a^	17.02 ^a^	13.22 ^b^	0.67	<0.0001	<0.0001	0.223
Butyrate	14.03	13.86	11.73	1.65	0.558	0.337	0.634
Isobutyrate	1.31	1.67	1.59	0.23	0.517	0.399	0.440
Valerate	3.15 ^b^	3.41 ^b^	4.69 ^a^	0.29	0.002	0.001	0.164
Isovalerate	2.75	3.25	2.61	0.52	0.670	0.853	0.387

CON, control diet; 2HILM and 4HILM are diets corresponding to 2% and 4% HILM inclusion levels, respectively; SEM, standard error of the means; BCFA, branched-chain fatty acid; SCFA, short-chain fatty acid. ^1^ BCFAs were isobutyrate, valerate, and isovalerate. ^2^ Total SCFAs were acetate, propionate, butyrate, isobutyrate, valerate, and isovalerate. ^a,b^ Values with different superscripts in the same row are significantly different (*p* < 0.05).

**Table 5:** Effect of the dietary *Hermetia illucens* larvae meal (HILM) inclusion level on the quality of the eggs.

Physical Attribute	Dietary Treatment			SEM	*p*-Value		
	CON	2HILM	4HILM		Diet	Contrast Analysis	
						Linear	Quadratic
Eggshell strength, kgf/cm^2^	5.30	5.14	4.96	0.27	0.674	0.381	0.969
Eggshell thickness, mm	0.42	0.43	0.43	0.01	0.678	0.450	0.661
Eggshell color, fan	12.75	13.06	13.56	0.45	0.444	0.212	0.865
Yolk color, fan	6.00 ^b^	6.06 ^ab^	6.50 ^a^	0.14	0.037	0.019	0.281
Haugh unit	89.03	84.75	85.33	1.53	0.124	0.102	0.209

CON, control diet; 2HILM and 4HILM are diets corresponding to 2% and 4% HILM inclusion levels, respectively; SEM, standard error of the means. ^a,b^ Values with different superscripts in the same row are significantly different (*p* < 0.05).

**Table 6:** Effects of the dietary *Hermetia illucens* larvae meal (HILM) inclusion level on the proximate composition and cholesterol content of eggs.

Item	Dietary Treatment			SEM	*p*-Value		
	CON	2HILM	4HILM		Diet	Contrast Analysis	
						Linear	Quadratic
Water, %	75.72	76.11	77.61	0.71	0.166	0.075	0.533
Protein, %	12.85	12.71	12.71	0.11	0.624	0.405	0.629
Lipids, %	9.80	9.66	9.82	0.13	0.658	0.921	0.369
Ash, %	1.00	1.10	1.02	0.04	0.277	0.858	0.116
Cholesterol, mg/100 g	368.33	365.77	342.75	7.88	0.061	0.032	0.301

CON, control diet; 2HILM and 4HILM are diets corresponding to 2% and 4% HILM inclusion levels, respectively; SEM, standard error of the means.

**Table 7:** Effect of the dietary *Hermetia illucens* larvae meal (HILM) inclusion level on the fatty acid profile (percentage of total fatty acid methyl esters) of laying hen eggs.

Item	Dietary Treatment			SEM	*p*-Value		
	CON	2HILM	4HILM		Diet	Contrast Analysis	
						Linear	Quadratic
Fatty acids							
C14:0 (Myristic)	0.29 ^b^	0.33 ^ab^	0.37 ^a^	0.02	0.005	0.001	0.947
C16:0 (Palmitic)	23.98 ^b^	25.17 ^a^	24.84 ^ab^	0.29	0.022	0.045	0.042
C17:0 (Magaric)	0.21	0.19	0.19	0.01	0.110	0.091	0.196
C18:0 (Stearic)	9.88 ^a^	9.36 ^ab^	8.91 ^b^	0.25	0.037	0.011	0.908
C23:0 (Tricosanoic)	0.12	0.13	0.13	0.01	0.764	0.498	0.793
Total SFA	34.48	35.18	34.44	0.25	0.080	0.915	0.026
C16:1 *n*-7 (Palmitoleic)	1.79	1.99	2.04	0.09	0.142	0.065	0.484
C18:1 *n*-9 (Oleic)	39.91 ^b^	40.98 ^ab^	42.55 ^a^	0.61	0.020	0.006	0.743
C20:1 *n*-9 (Eicosenoic)	0.18	0.18	0.19	0.01	0.215	0.214	0.210
Total MUFA	41.88 ^b^	43.15 ^ab^	44.78 ^a^	0.61	0.010	0.003	0.813
C18:2 *n*-6 (Linoleic)	18.14 ^a^	17.67 ^ab^	17.08 ^b^	0.62	0.019	0.007	0.362
C18:3 *n*-6 (γ-Linolenic)	0.12	0.12	0.11	0.01	0.158	0.077	0.452
C18:3 *n*-3 (Linolenic)	0.63	0.61	0.60	0.03	0.088	0.045	0.341
C20:2 *n*-6 (Eicosadienoic)	0.15	0.15	0.14	0.01	0.110	0.051	0.435
C20:4 *n*-6 (Arachidonic)	1.73	1.77	1.70	0.03	0.228	0.383	0.138
C22:6 *n*-3 (Docosahexaenoic)	0.72 ^ab^	0.75 ^a^	0.67 ^b^	0.02	0.033	0.042	0.080
Total PUFA	23.13 ^a^	21.06 ^ab^	20.29 ^b^	0.67	0.020	0.007	0.443
UFA/SFA	1.89	1.83	1.89	0.02	0.054	0.897	0.017
*n*-6	21.70 ^a^	19.70 ^ab^	19.03 ^b^	0.64	0.020	0.008	0.410
*n*-3	1.43 ^a^	1.36 ^ab^	1.27 ^b^	0.04	0.043	0.014	0.816
*n*-6/*n*-3	15.23	14.50	15.03	0.31	0.254	0.668	0.113

CON, control diet; 2HILM and 4HILM are diets corresponding to 2% and 4% HILM inclusion levels, respectively; SEM, standard error of the means; SFA, saturated fatty acid; MUFA, monounsaturated fatty acid; PUFA, polyunsaturated fatty acid; UFA, unsaturated fatty acid. ^a,b^ Values with different superscripts in the same row are significantly different (*p* < 0.05).

**Table 8:** Heavy metal concentration (mg/kg) in *Hermetia illucens* larvae meal (HILM).

Item	HILM	Permissible Limit [26]
Essential elements		
Mg	5600	NA
S	562.24	NA
Essential trace elements		
Fe	560.90	NA
Zn	142.15	NA
Cu	456.34	NA
Cr	59.06	NA
Co	4.51	NA
Se	0.48	NA
Mn	101.08	NA
I	ND	NA
Undesirable substances		
Al	3.25	NA
F	0.01	150
As	0.01	2
Pb	0.01	10
Hg	<0.01	0.1
Cd	ND	2

NA, not applicable; ND, not detected.

**Table 9:** Heavy metal concentration in the eggs of laying hens that fed on *Hermetia illucens* larvae meal (HILM).

Item	Dietary Treatment			SEM	*p*-Value			Permissible Limit
	CON	2HILM	4HILM		Diet	Contrast Analysis		
						Linear	Quadratic	
Essential elements								
Mg, mg/kg	114.66 ^b^	118.79 ^b^	128.87 ^a^	2.75	0.004	0.002	0.387	NA
S, %	0.20	0.20	0.20	0.002	1.000	1.000	1.000	NA
Essential trace elements, mg/kg								
Fe	16.92	17.99	20.00	1.14	0.174	0.069	0.738	NA
Zn	11.40 ^b^	13.33 ^b^	17.05 ^a^	0.81	0.0003	<0.0001	0.379	NA
Cu	<0.10	<0.10	<0.10	-	-	-	-	NA
Cr	<0.10	<0.10	<0.10	-	-	-	-	NA
Co	<0.10	<0.10	<0.10	-	-	-	-	NA
Se	<0.10	<0.10	<0.10	-	-	-	-	NA
Mn	<0.10	<0.10	<0.10	-	-	-	-	NA
I	ND	ND	ND	-	-	-	-	NA
Undesirable substances, mg/kg								
Al	1.34 ^b^	1.61 ^b^	3.58 ^a^	0.30	<0.0001	<0.0001	0.033	30.00 [27]
F	<0.01	<0.01	<0.01	-	-	-	-	NA
As	<0.01	<0.01	<0.01	-	-	-	-	0.04 [28]
Pb	<0.01	<0.01	<0.01	-	-	-	-	0.20 [29]
Hg	<0.01	<0.01	<0.01	-	-	-	-	0.05 [29]
Cd	<0.10	<0.10	<0.10	-	-	-	-	0.05 [29]

CON, control diet; 2HILM and 4HILM are diets corresponding to 2% and 4% HILM inclusion levels, respectively; SEM, standard error of the means; NA, not applicable; ND, not detected. ^a,b^ Values with different superscripts in the same row are significantly different (*p* < 0.01).

## Data Availability

The data presented in this study are available on reasonable request from the corresponding author.
